# Quiet as an Environmental Value: A Contrast between Two Legislative Approaches

**DOI:** 10.3390/ijerph10072741

**Published:** 2013-07-03

**Authors:** Robert Thorne, Daniel Shepherd

**Affiliations:** 1 Noise Measurement Services Pty Ltd, Brisbane 4051, Australia; 2 Department of Psychology, Auckland University of Technology, North Shore Auckland 0627, New Zealand; E-Mail: daniel.shepherd@aut.ac.nz

**Keywords:** amenity, environmental legislation, environmental values, quiet, soundscape, wellbeing

## Abstract

This paper examines the concept of “quiet” as an “environmental value” in terms of amenity and wellbeing from a legislative context. Critical review of two pieces of environmental legislation from Australia and New Zealand forms the basis of the paper. The Australian legislation is Queensland’s Environmental Protection Act, and the New Zealand legislation is that nation’s Resource Management Act. Quiet is part of the psychoacoustic continuum between a tranquil and an intrusively noisy sound environment. As such, quiet possesses intrinsic value in terms of overall sound within the environment (soundscape) and to individuals and communities. In both pieces of legislation, guidance, either directly or indirectly, is given to “maximum” sound levels to describe the acoustic environment. Only in Queensland is wellbeing and amenity described as environmental values, while in the New Zealand approach, amenity is identified as the core value to defend, but guidance is not well established. Wellbeing can be related to degrees of quietness and the absence of intrusive noise, the character of sound within an environment (“soundscape”), as well as the overall level of sound. The quality of life experienced by individuals is related to that person’s physical and mental health, sense of amenity and wellbeing. These characteristics can be described in terms of subjective and objective measures, though legislation does not always acknowledge the subjective.

## 1. Introduction

### 1.1. Quiet: A Human Perception of Soundscapes

The expression “peace and quiet” is often given as the benchmark for amenity in relation to sound in an environment (*i.e.*, soundscapes), but what is meant by peace, quiet, amenity and noise can vary across contexts, and these expressions can mean different things to different people at different times. An example is the absence of an explicit definition of what constitutes a quiet area in the European Noise Directive (END) approach [1], although others have attempted definitions [2,3]. Typically, values for quiet in urban and rural locales are defined only in terms of sound level [4,5] or distance to major transportation activity (road, rail, aircraft) or industry [6,7,8]. The approach adopted under END is through strategic noise mapping, although some authorities are attempting to combine environmental quality objectives (e.g., [9,10]). The concept of a tranquillity rating [11] provides a methodology to evaluate a locale with parks and gardens in terms of their “restorative” or “tranquil” capacities, and it would likely be possible to combine this approach with the END’s noise maps in future.

Booi and van den Berg [8] investigated an urban situation in order to assess (amongst other matters) what constitutes a quiet place and to what extent residents want peace and quiet. Their conclusion is worth presented verbatim:

*“The need for quietness is strongly related to noise sensitivity and the perception of sound. When sound is perceived as a negative factor (noise from transportation and people) there is a higher need for quietness, but a positive factor (perceived liveliness at home/in neighbourhood) it reduces that need.”*

*“It is clear that the acoustic quality of the city not only depends on the absence of noise but also on the presence of quietness and liveliness. … A city can be very noisy, but that is less a problem if its inhabitants have access to quiet places: a quiet home and a quiet place outdoors”.*

Furthermore, Booi and van den Berg [8] suggest that, for an urban environment, *“…areas can be considered to be quiet at sound levels due to road and rail traffic up to 60 dB L_day_, a level that is known to cause indoor noise annoyance in a part of the population.”* In contrast, the study of rural environments in Ireland [9] suggests significantly lower sound levels are required for Quiet Areas: *“noise from anthropogenic sources should not be clearly audible at any point within Quiet Areas… should not exceed an L_A90,1h_ of 30 dB by day or an L_A90,1h_ of 27 dB by night”* (note: *L_day_* is defined in [1] and *L_A90,1h_* is the background sound level).

The protection of quiet areas, a core objective of the END [1], is an important, but often overlooked, activity given their contribution to viability. People relate to environments on an emotional level by interpreting the sensory information afforded by their landscape including the character of sound within the environment. This is a quality of life relationship, where general pleasurable sounds promote, and annoying sounds impede, health [12,13,14]. The wide variation in noise sources and their audible characteristics are closely linked to noise annoyance and definition of soundscapes [15,16,17]. Soundscape can be measured in both subjective and objective terms [18,19,20]. A new International Standard ISO 12913 [21] is proposed to address the definition of “soundscape” and general concepts and relationships: (a) sound sources, (b) acoustic environment, (c) auditory sensations, (d) interpretation of auditory sensations, (e) responses, (f) context, and (g) outcomes. The perception of the sounds or noise in the environment and a person’s relationship to both the noise and the environment is critical. Broadly, this can be defined as a person’s sense of amenity with respect to environmental noise.

### 1.2. Amenity: A Sense of Place

Generally, amenity values are based upon how people feel about an area, its pleasantness or some other value that makes it a desirable place to live [22,23]. In particular, landscape and soundscape characteristics mark an environment as a desirable (high amenity) or undesirable (low amenity) place to occupy, as uniquely judged by the individual. Localities hosting stressors tend to provoke negative emotions, and motivate an avoidance response, while localities free from stressors may induce positive emotions and motivate an approach response. Generally, people are motivated to seek places offering the greatest level of amenity, that is, that minimize stress, maximize restoration, and induce a positive sense of wellbeing. As a consequence, community groups, town planners and architects work to provide high amenity areas in terms of landscapes and soundscapes, typically parks or natural/wilderness areas that are often described by visitors as being “peaceful”, “quiet” or “tranquil” (e.g., [9,24]). Quality of life [25,26] therefore provides an essential factor in determining amenity, not just or solely the physical components of amenity as given in New Zealand and Australian state of Queensland environmental legislation:

(a) *Amenity values*: those natural or physical qualities and characteristics of an area that contributes to people’s appreciation of its pleasantness, aesthetic coherence, cultural and recreational attributes [27].
(b) *Community amenity*: the qualities of the acoustic environment that are conducive to protecting the amenity of the community [28].

The second of these definitions, from Queensland, is somewhat circular in nature and is described later in this paper in relation to a Planning and Environment court decision [29] dealing with amenity. In both countries the context of the legislation is to reduce or minimise the adverse effects of noise, in order to preserve amenity and wellbeing. In a legislative sense, the definition of amenity is stricter and often occurs in the framework of property law. For example, the 9th Ed (2009) of Black’s law dictionary [30] defines amenity thus:

*“In real property law. Such circumstances, In regard to situation, outlook, access to a water-course, or the like, as enhance the pleasantness or desirability of an estate for purposes of residence, or contribute to the pleasure and enjoyment of the occupants, rather than to their indispensable needs. In England, upon the building of a railway or the construction of other public works, “amenity damages” may be given for the defacement of pleasure grounds, the impairment of riparian rights, or other destruction of or injury to the amenities of the estate. In the law of easements, an “amenity” consists in restraining the owner from doing that with and on his property which, but for the grant or covenant, he might lawfully have done; sometimes called a “negative easement” as distinguished from that class of easements which compel the owner to suffer something to be done on his property by another.”*

and as expressed in Chapman v. Sheridan-Wyoming Coal in 1950 [31]:

*“Amenity is something on the attractive, pleasant or desirable side of life. In legal parlance, amenity is the location, view, access to water courses or lakes, etc. which add to the desirability of a tract of real estate. When a right consists in restraining the owner from doing that with, and upon, his property which, but for the grant or covenant, he might lawfully have done, it is an amenity or negative easement*.”

In New Zealand the concept of “reverse sensitivity” has evolved through case law, and applies where new sensitive activities are introduced into an environment where lawfully established activities may create adverse effects upon the new activity. The physical amenity values of all parties are considered but quality of life is not, unless it can be defined in terms of “pleasantness”. In the south of New Zealand, for example, the Clutha District Council in its District Plan considers noise thus:

*“The Resource Management Act requires Council to “control the emission of noise and the mitigation of the effects of noise”. Noise pollution creates an all invading source of intrusion into an environment and can adversely affect people’s wellbeing. These effects are often hard to define precisely but regard must be had to the incidence of noise from any activity.”*

It has long been noted that the transformation of sound to noise, and thus the assessment of “intrusive” noise, or “nuisance” noise, depends upon individual sensitivity to the noise and the context in which the sound occurs. Intrusive noise and/or annoyance [32,33,34] can be defined in terms of impact, referenced to before, during and after some identified noise event. Reaction modifiers (e.g., [35,36,37]) for individuals include: (a) attitude to noise source, (b) attitude to information content in the noise, (c) perceived control over the noise, (d) sensitivity to noise (in general and specific measures), and (e) sensitivity to specific character of the noise (e.g., changes in pitch or modulation). These reaction modifiers can be integrated into definitions for intrusive sound, namely noise and intrusive noise, that can be applied in the legal context, and that allow qualification (*i.e.*, subjective) and quantification in physical units (*i.e.*, objective) (e.g., as described in [4,10,18]):

*“Noise* is a sound that is perceptible to an individual and has definable characteristics that modify the individual’s emotional and informational responses to that sound from pleasurable or neutral to adverse.”

*“Intrusive noise*, to an individual, is a sound whose character (such as audibility, dissonance, duration, loudness, tonality, pitch or timbre) is perceived adversely compared to the character of the environment in the absence of that sound.”

These two definitions of noise can undergo synthesis and a rank order of impact derived thereof. However, it should be remembered that there is no objectively defined relationship that can predict when environmental values and perceived amenity may be adversely affected by noise. Regardless, at the experiential level, sound is commonly judged as being reasonable or unreasonable, wanted or unwanted, pleasant or annoying, easy to ignore or intrusive.

### 1.3. Wellbeing: A Determinant of Good Health

Wellbeing is a defined determinant of population health under the WHO strategy “Health for all in the 21st century” [13]. The legislation described in this paper present environmental protection variables that are amenable to public policy and where changes are designed to improve health and wellbeing for the whole population. The Queensland noise policies, for example, apply both Target 10 “A healthy and safe living environment” and Target 11 “Healthier Living” by adoption of environmental values to be enhanced or protected and acoustic quality objectives to be applied to manage noise in the environment.

Wellbeing and amenity can therefore be qualified and quantified in terms of “quiet” and “noise” (as defined in [33]):

(1) *No adverse effect*, pleasurable sounds or peace and tranquillity; quietness
(2) *Minor adverse effect, minor irritation*; minor intrusion of noise on occasion external to the home, no modulation or distinctive tonality
(3) *Adverse effects more than minor*; intrusive noise audible on occasion within the home, no modulation or distinctive tonality
(4) *Nuisance adverse effect*; intrusive noise heard within or exterior to the home on a regular or definable basis, modulation or distinctive tonality may be present; causing anger, annoyance, or adverse health reactions including sleep disturbance
(5) *Significant adverse effect*; irrespective of sound character causing annoyance or anger and or adverse health reactions including sleep disturbance.

Based on the foregoing: criterion (1) infers no noise whatsoever; criterion (2) is “reasonable noise”; criterion (3) is the transition stage between unreasonable and reasonable noise; and criteria (4) and (5) define “unreasonable noise”.

### 1.4. The Economics of Quietness

The impact on sound upon amenity can be indirectly estimated using economic measures. In Australia, historical data from the 1986 national noise survey [38], and in Queensland specifically, the 1988 Brisbane noise survey [39], both reflect the high value that people place on a quiet neighbourhood and the amenity they afford. The Brisbane survey reported that 17 percent of respondents had considered moving because of noise, and 55 percent thought that noise adversely affects the value of their property. The effect of noise on lifestyle has been qualified and quantified to a degree [40]. However, further quantification, such as determining what value to place on non-traded commodities such as access to quiet areas for recreation purposes, is a more complex issue. Conventional amenity valuation by economists aims to measure the willingness of people to pay for a quiet environment compared with their willingness to pay for noise control services, such as entertainment noise control. This valuation can also relate to the concepts of environmental values and quality of life [41].

The valuation of quiet or noise as commodities is not an untoward concept. They are commodities that can be bought and sold like any other commodity. As there is not an accepted system for the definition of cost of noise mitigation to achieve regulatory goals, criteria need to be defined for the quantification of value in dollar terms [42]. Typically, peace, tranquillity and quiet have value while noise has cost. This is because noise affects individuals and communities by modifying the extrinsic and intrinsic nature of the environment that attracts and holds people to the locality. The noise may have a positive value (e.g., a “nil-delivery” warning beeper on a cardiac patient’s oxygen system) or, more likely given its nature, a negative value (e.g., high levels of tonal noise from an industrial activity). Unregulated noise emissions—“immissions”, for example, impose a cost on to the receiver of that noise, without compensation or redistribution of cost back to its creator. There is a cost in producing the noise, a cost in receiving the noise, and a cost in reducing or mitigating such noise. However, while noise can typically be quantified by sound exposure levels or audibility using scientific equipment, and qualified in terms of unwantedness, annoyance and loss of amenity using self-report measures, the economic consequences of noise are not so easily estimated.

Although economic factors are major determinants in the choice of neighbourhood, both lifestyle and environmental conditions tend to be strong influences on the actual locations chosen. The movement of people to areas of environmental advantage is an important factor in planning considerations. Given a favourable economic setting, regions with high environmental aesthetics are likely to exhibit better economic performance than regions with environmental problems. In this respect, noise mitigation becomes a social process, whereby individuals invest personal resources into quiet areas, and in doing so, lower demand for areas considered noisy. However, caution is needed when assessing noise sources and levels, due to the extrinsic and intrinsic nature of the receiving environment. For example, concepts such as “sense of place” [22] need to be accounted for in green areas, but less so in urban areas.

## 2. Soundscape Analysis in Australia and New Zealand

The impact of noise on communities and characterisation of soundscapes has been mostly studied in European contexts. In other parts of the world, such as Australia and New Zealand, noise data is impoverished, and noise policy often guided by data collected in other countries (e.g., [43]). The lack of attention to the quantification of impact in specific noise areas is regrettable, as noise is the most widely reported form of pollution affecting communities. In Queensland, Australia, 22 percent of Queensland participants in a 1986 national study reported disturbance by noise at some time [38]. Furthermore, the survey indicated that residents regard noise pollution as one of the most serious forms of environmental pollution within their homes, with traffic noise, barking dogs and lawn-mowers causing the most irritation to people. The later 1988 study [39] in the Australian City of Brisbane’s metropolitan area also indicated that residents were more concerned about noise pollution than any other form of urban pollution. This study found that road transport noise was the noise source of greatest concern to residents, with 35 per cent of respondents claiming to be “seriously affected” by noise from light vehicles and 25 per cent by heavy vehicles. In fact, noise is responsible for the majority of all complaints received by Queensland’s Department of Environment and Heritage Protection, which has legislative oversight of the environment in relation to human activity and biodiversity.

A conservative estimate of the percentage of the Australian population affected by excessive noise comes from Brown [40]. He found that over 9 percent of the Australian population is exposed to traffic noise greater than 68 dB(A), and 19 percent of the population is exposed to 63 dB(A) or above. For all urban centres exceeding 100,000 residents, it is estimated that only 42 percent will have an acceptable level of 55 dB(A) or less. An alternative estimate of traffic noise exposure is given by Modra [44] who estimated that, on average, each kilometre of major road in the Australian City of Melbourne fronted 57 houses or other dwellings, and that 81 percent of these residences were exposed to noise levels greater than 68 dB(A). Assuming that these estimates hold true for Brisbane, the 620 km of major roads in the Brisbane metropolitan region would result in approximately 28,000 houses or an estimated 84,000 people being exposed to noise levels above 68 dB(A). This represents 5.2 percent of the city’s residents. Thus the prevalence of noise annoyance, and its increasing incidence, is of concern because, as the literature indicates, noise negatively impacts health and wellbeing. In more detailed European analyses, the WHO [45] conservatively estimated that 587,000 DALYs (disability-adjusted life years) are lost due to noise-induced annoyance within European cities and, 903,000 years for sleep disturbance. The WHO’s “Health 21” strategy [13] contains specific targets to deliver healthy and safe environments (Target 10) and sustainable health (Target 11).

New Zealand noise studies, of which few exist (but see [26,46]), suggest that noise constitutes a most serious neighbourhood problem [47], and a “reality television” show (“Noise Control”) has been aired on a national television channel documenting noise-related neighbourhood conflicts [48]. One recent study (Welch *et al.*, in press [49]) found that 11.9% of a large urban sample indicated that they were highly annoyed by road traffic noise, with those living in close proximity to a main road (24.9%) more likely to report high annoyance than those living further away (4.7%). An earlier study [46] reviewed ambient sound levels in rural and residential areas, industrial and commercial locales and transport (road, rail, airport) networks. Quiet was a desired individual good in all localities. The overall responses from the study indicated that of the persons that had an opinion, 24% were affected while reading, 24% while watching TV, 29% while in conversation, 40% while relaxing, and 29% while sleeping (or attempting to get to sleep).

## 3. Queensland’s Approach to Amenity and Wellbeing

### 3.1. The Concept of “Environmental Values”

The concept of community and individual “environmental values” is a relatively recent legislative construct, with the notion of “amenity” becoming a necessary, albeit difficult, environmental value needed to protect human health and wellbeing. In Queensland (Australia), the Environmental Protection Act 1994 [50] established a legal mechanism to recognise environmental values important to the community, while the Environmental Protection (Noise) Policy 1997 [42,51,52] identified the acoustic features of environments to be enhanced or protected. Such qualities of the acoustic environment necessitating guardianship are those that are conducive to: (a) the wellbeing of the community, including social and economic wellbeing, and (b) the health and wellbeing of an individual, including the individual’s opportunity to have sleep, relaxation and conversation. Redrafted in 2008 [28] the revision re-emphasizes the protection of human health and wellbeing, as well as protecting environmental amenity and the wellbeing of individuals and the community. The 2008 policy extends the environmental values to protect the health and biodiversity of ecosystems. The wellbeing of a community is an environmental value established in the 1997 Policy, and updated by the 2008 Policy, as “the qualities of the acoustic environment that are conducive to protecting the amenity of the community”. This is a somewhat circular definition in respect to amenity, but quantifiable and measurable nonetheless. In some cases (e.g., mining in a greenfields environment), an anthropogenic analysis of the qualities of the existing and future (affected) acoustic environment is necessary for a complete risk assessment. The wellbeing of individuals is identified in both the 1997 and 2008 policies. The 1997 Policy established the concept of an “acoustic environment” with an ambient sound level of 55 LAeq (24 h) and the provision that the existing environment not be allowed to deteriorate. This is an objective soundscape measure to complement the wellbeing of the community and individuals.

The Queensland legislation (1997/2008) demonstrates that environmental qualities affecting both human and ecosystem (biodiversity) health can, therefore, be defined in an effective and practical manner in different legislative frameworks relating to amenity.

The 2008 Environmental Protection (Noise) Policy established a range of “acoustic quality objectives”. The acoustic quality objectives, based on WHO criteria [43], are “maximum” sound levels that should be experienced in the acoustic environment of an area or place. They are soundscape values and define measures of “Quiet” to “Loud”. The environmental values to be enhanced or protected under the policy are the qualities of the acoustic environment that are conducive to protecting the health and biodiversity of ecosystems; the qualities of the acoustic environment that are conducive to human health and wellbeing, including ensuring a suitable acoustic environment for individual’s to sleep, study and learn, to be involved in recreation including relaxation and conversation, and; the qualities of the acoustic environment that are conducive to the protection of individual and community perceptions of amenity. The objectives are read in association with the environmental values just described. To date, no environment court cases in Australia have been heard to provide guidance as to the relationship between the environmental values, acoustic quality objectives, nuisance, environmental harm, amenity and wellbeing. The acoustic quality objectives are maximum values, however, they do not reflect the current state of knowledge relating to the human perception of low frequency sound, nor the potential effects of noise on non-human ecosystems. The Explanatory Notes [53] to the Policy state:

*“It is not intended that, as part of achieving the acoustic quality objectives, any part of the existing acoustic environment be allowed to deteriorate. (p. 9)”*

*“The acoustic quality objectives are to inform the decision making process including any conditions relating to noise levels in relation to the decision. The objectives assist in identifying whether the environmental values are protected. However, meeting the objectives does not always mean that the environmental values are protected and not meeting the objectives does not always mean that the environmental values are not protected. (p. 10)”*

*“The acoustic quality objectives are levels of total noise, which means the objectives include the surrounding noise associated with any given environment and therefore includes the range of noise that may be experienced in the environment. (p. 10)”*

### 3.2. Amenity—Analysis and Examples of Application

Arguably, the amenity of the acoustic environment relative to a specific community requires a more finely detailed analysis than the “maximum” sound levels expressed by the acoustic quality objectives. Consequently, the objectives must be considered in relation to what they are seeking to achieve and their relationship to the relevant environmental values. These, in turn, require some form of analytical methodology in order to provide context to subjective and objective measures. Indeed, given the differences in approach to the definition of soundscapes and environmental noise [24], criteria based on audibility maybe more appropriate although difficult to implement.

Activity disturbance is regarded as an important indicator of the impact of noise on the community, as outlined in Section 7(b) of the Queensland legislation (2008) protecting the right to relaxation and unhindered conversation. According to the 1986 national noise survey, disturbances were to listening activities (e.g., listening to the radio or watching television) and sleep disturbance. Overall, 40 percent of the respondents reported disturbance to listening activities (traffic noise 13 percent, barking dogs 8 percent) and 42 percent reported disturbance to sleep (barking dogs 15 percent, traffic noise 12 percent). However, it should be noted that noise masks not only activity sounds (e.g., conversation/TV) but also ambient sounds that may be otherwise restorative (waves at the beach, birdsong, children playing *etc.*), an impact of noise not considered in the Queensland legislation (1997/2008).

Actual court cases that have addressed amenity values in the Queensland context are not common. In 2012 the Land Court of Queensland [54] heard a case in relation to mining and amenity from people living in the proximity of a proposed open-cut coal mine. The applicants submitted that the quiet enjoyment of a resident’s land would not be affected by altering the boundary of the mining lease and the resident’s complaint was really one of compliance with noise conditions. The question of degraded amenity was discussed in the context of background sound levels and levels of noise that are likely to cause sleep disturbance. The noise assessment was initially carried out with reference to the Queensland 1997 noise policy and a later assessment applied the 2008 noise policy and acoustic quality objectives. The decision, included issues other than noise, confirmed a draft noise limit condition of 35 dB(A) LA_eq_ measured over 15 min outside the dwelling in order to achieve a sound level at night of 30 dB(A) LA_eq_ inside a bedroom. The bedroom level, however, was measured over one hour with a provision of penalties for tonality or impulsiveness. The Land Court decision is useful in the context of discussing, very briefly, the application of the noise policy and the application of noise numbers to define or establish sleep disturbance. Unfortunately, the Court’s decision does not give any guidance to the actual quality and potential for noise to affect local ecosystems, for example, the territorial boundaries of local bird life.

The effect of noise on ecosystems is nearly always overlooked in an acoustic assessment for large-scale activity in a greenfield location. Bird-life mark their territories for food and mating through bird-song and calls. Any human intervention that disrupts this cycle is potentially reducing the diversity in an ecosystem by removing a predator group and allowing the growth of the food group. This can have unintended consequences through the food chain. The strict application of noise-numbers, as given in the noise policy, fails to identify, qualify or quantify the effects of noise on ecosystems including human ecosystems. A risk-assessment approach is needed, with the environmental values having primacy and the acoustic quality objectives being identified as simplistic noise-numbers for design.

A different approach to the consideration of amenity was given by the Planning and Environment Court of Queensland in the case of Holcim (Australia) PL v Brisbane City Council [55]. This case involved a residential development proposed in the near vicinity of existing industry: a concrete batching plant. The Court said that the amenity of the residential development must not be affected by the operation of the concrete batching plant and the planning provisions were:

*“…clearly designed to guard against any adverse effect on the amenity of any new residential development which results from the concrete batching operations.”*

The Council had confined its focus to the question of amenity due to air quality, noise and traffic. The Court held that the Council was obligated to properly address the entire concept of amenity. It did not do so and the approval for the residential development was set aside. This decision is significant because the Court has clearly indicated that “amenity” is not a simple assessment against set criteria but encompasses a diverse range of perceptual values. The Court referred to amenity and quiet by reference to an earlier decision [29]:

*“…Amenity in its most obvious and narrow sense might be related to such physical features as sight, sound and smell. In a broader sense, however, it relates to more illusory concepts. To take example suggested by Mr. Cooke, Q.C., for the Appellant, a small funeral parlour might be designed to look like a house. It might have a landscaped, hidden parking area, with a discreet access point so that traffic to and from it is barely noticeable. It might be quiet by day and absolutely silent by night. Yet it would have an unmistakable—air or—feel to it which would have an adverse effect on a residential amenity.”*

The decision is an example where perception was taken as being of greater import or value than some form of objective measurement. This was not a case of where the noise from the industry could be managed, or the residences built to reduce noise inmission, but a subjective opinion that the amenity of the development as such was adversely affected to the extent that the development could not proceed.

## 4. New Zealand’s Approach to Amenity

### Measures for Amenity

Though close geographically, and much legislation and practices are combined across the two nations, Australia and New Zealand manage the acoustic environment using different approaches. In New Zealand, neighbourhood noise is policed on a regular basis by local government rather than a police function as in Australia, and lacks a unified noise policy such as Queensland’s, with at least five legislative acts (Dog Control Act 1996; Traffic Regulations Act 1976; Health and Safety in Employment Act 1992; Residential Tenancies Act 1996; Resource Management Act 1991) and individual Council planning rules involved in the management of environmental noise. Of these, however, it is the Resource Management Act [27] that serves to guide national and local authorities on both acceptable noise levels and the abatement of unacceptable noise levels. The RMA provides a legal framework for noise control by defining amenity values and guarding against “unreasonable” noise (Section 16) and “excessive” noise (Section 326).

The RMA, the centre piece of New Zealand’s environmental policy, contains no reference to “quiet”, and provides no quantitative criteria of “excessive” noise levels, instead directing local authorities to environmental (noise) standards when considering permissible noise levels in their district plans. Qualitatively, “excessive” noise is defined in the RMA as sound that is under human control, including musical instruments, electrical appliances, machines (however powered), a person or groups of persons, or explosions or vibrations. Excluded from this definition is noise emanating from aviation, rail, or road transportation. More specifically, the RMA notes that excessive noise “unreasonably interferes with the peace, comfort, and convenience of any person (other than a person in or at the place from which the noise is being emitted)”. However, “unreasonably interferes” carries the implication that noise can “reasonably interfere”. In practice, day-by-day guidance as to what is, and what is not, “reasonable noise”, is one-or-other of the environmental noise standards, which themselves borrow heavily from international standards (e.g., WHO Noise Guidelines) focusing on health impacts rather than amenity. Thus, *prima facia*, the RMA ultimately refers decision makers to a definition of noise that can be traced back to the New Zealand’s Health Act (1956: Section 23, Paragraph K only): “…where any noise or vibration occurs in or is emitted from any building, premises, or land to a degree that is likely to be injurious to health.”. The emphasis on health may not initially appear consistent with the more amenity-based criteria of “peace, comfort, and convenience” explicitly referred to in the RMA, but reference to the World Health Organisation’s definition of health (1948): “Health is a state of complete physical, mental and social wellbeing and not merely the absence of disease or infirmity.” indicates that health is not defined only by morbidity or mortality, but other factors such as environmental amenity. In reality, however, health-based noise standards such as those developed by the WHO focus primarily on sleep disturbance and cardiovascular function, while measures more sensitive to effects on amenity, such as annoyance, to a much lesser extent.

The national environmental standards, including noise standards, are developed by Standards New Zealand, a government owned but independently operated organisation. New Zealand noise standards include NZS6801/6802 (measurement and assessment of sound/noise), NZS6803 (transport noise), and NZS6808 (wind turbine noise). Through these noise standards, environmental noise assessment in New Zealand consists, to a large degree, of the management of sound from various sources referenced to some pre-determined “baseline” sound level. This level may be daily exposure (such as the United States’ day-night level or the EU day-evening-night level) or for some shorter or longer period of time. Implicit in these assessments is that a certain proportion of the community will be highly annoyed by the source of noise at a nominated sound level threshold. This proportion is in the order of less than 20 percent of the exposed population, depending on the source of noise and the baseline sound level [25]. New Zealand district plans often refer to some statistical sound measure but there is no evidence that, for example, an external level of 45 dB(A) L_10_ is an acceptable or desirable night-time upper limit at a dwelling. Similarly, there is no evidence from extensive acoustical and attitudinal surveys in New Zealand (734 households and 1225 individual responses, [46] that 55 dB(A) L_10_ is an acceptable or desirable daytime upper limit. These limits are those recommended in many New Zealand District Plans and various New Zealand standards for environmental noise (e.g., NZS6801:2008 and NZS6802:2008), and unfortunately have a very weak scientific justification.

As described, New Zealand has a different approach for activities that require planning approval, which come under the RMA. The RMA approach, through the environmental standards, is based upon an analysis of the nature of the sound source, the nature of the receiving environment and the potential for adverse effect due to the sounds (see, for example, [26]). However, the RMA process appears to have application only when an activity is referred to the Environment Court for decision, an option that is often beyond the financial reach of noise-impacted individuals or communities. Experience within the Environment Court process indicates that this approach does not seek to protect environmental values but rather judge acceptable deviations of individual amenity from the pre-determined baseline amenity for the community-at-large. The RMA gives a definition of “amenity values” that is very similar to the intent of the Queensland legislation. The RMA definition, as defined above in Section 1.1, implies that it is insufficient to rely on noise-numbers as the sole determinant of environmental values or amenity; human perception of the environment is a critical factor. Some guidance with respect to amenity is available from the Ministry for the Environment (see, for example, [56]). The guide notes that *“RMA plans have to manage the effects of activities”* and *“Resource users should confine their* *adverse activities within their own sites. Adjoining owners should not be expected to suffer adverse effects”.*

The practice, however, is completely different as experience before the Environment Court individually by the authors has shown. Noise performance standards and “noise-numbers” incorporated within district plans or New Zealand Standards are applied unless there is significant proven reason why some other indicator should be adopted. Consequently, individuals and local communities in New Zealand appear to have very little redress against noise from industrial activities affecting neighbouring properties unless such noise is so unreasonable or excessive that the territorial local authority is forced to take legal action for mitigation. In contrast, New Zealand has a very robust and effective control process for noisy parties or loud music within its urban limits.

An example of the New Zealand “noise mitigation” system in practise can be seen with the Te Rere Hau wind farm, located in New Zealand’s “wind province”, Palmerston North. After obtaining planning permission and becoming operational, a complaint of noise was heard by the Environment Court and the Court decided, amongst other things, that the noise effects at local residential locations are considerably greater than those predicted in the application. The process started by an application of the Palmerston North City Council to the Environment Court [57] in November 2010 on the grounds that the wind farm operator had significantly under-estimated the effects of the wind farm noise on the amenity of the area. The Council application provided grounds relating to both overall sound levels (soundscape) and “special audible characteristics” (human perception). While the process is taking a long time it does show that quiet, noise and amenity can be assessed under New Zealand’s legislative constructs, but at a large cost of time and money to the affected individuals or communities.

## 5. Applying the Different Approaches

Both the Australian and New Zealand approaches attempt to establish rules to redefine subjective experiences into quantitative measures. With suitable measures in place it is possible to objectively define the character of the ambient soundscape and intrusive sound in measurable terms. A fundamental difference between Queensland’s Environmental Protection Act and the New Zealand Resource Management Act is that the former is specifically designed to protect and conserve the environment, and amenity therein, while in practice the latter is a resource management and planning instrument that is not specifically designed to protect the environment, but rather manage the environment as a social or more usually, an economic resource.

 Acoustical analysis has little meaning to a person unless it is has a real relationship with an individual’s responses to intrusive sound and can be described or explained in a way that the individual understands. Individuals understand intuitively what “noise” is to them personally, and this distinction may change day-by-day, even to the same sound. Individually perceived amenity is assessed as an *intrinsic* value reflecting noise sensitivity, personal and cultural attitudes to sound in the environment, the environment itself, and habituation effects. The *extrinsic* values that affect perceived amenity are presented as community values that may have potential effect on the individual. These community values are often mistaken as applying to all individuals. It is this failure to understand that individuals are not responsive to “average-values” (*viz.* the ecological inference fallacy) that causes so much conflict between the identification of the sound/noise in the environment, people making noise and people exposed to noise.

In order to establish what is wanted as “Quiet” it is necessary to first establish the level and character of soundscape. This involves not only objective noise measurements obtained using instrumentation, but also the judgments and reactions of those occupying the soundscapes. If the character of the sound is foreign (*i.e.*, incongruent) to the existing environment then it has less chance of being accepted, and can come to dominate the soundscape if it directs attention. To an individual, the time of the day the sound is heard is important with unusual sounds in the early morning (e.g., at 2:00 a.m.) being less acceptable than if they are heard during daytime hours. Sounds that disturb sleep are nearly always unacceptable even if they have some potential benefit to the individual (e.g., smoke alarms). The number of times a sound is heard (*i.e.*, frequency of sound events) and the duration of the sound are important but, even though there are some studies for transportation noise they do not sufficiently investigate impacts of low amplitude sound. If a sound intrudes the personal space of a person while at home, inside or outside, that sound has a high probability of becoming a disturbance. Additionally, if the sound contains unwanted information then that sound will also be likely to be perceived negatively. Personal perception therefore combines a variety of attributes that cannot be measured by instrumentation, and this itself poses great difficulty at the legislative level.

In evaluating the “quality” of a sound, the audibility or perception of the sound is assessed by an individual who then express their judgements using different terms to describe the sound. An individual may describe in many different ways the perceived character of a sound, and the emotions it elicits. The techniques of sound quality measurement (e.g., loudness, dissonance) provide a partial analogy for sensitivity to noise.

While a critical part of the human perception of sound, sound quality measures are very difficult to incorporate as practical and effective objective criteria in standards or codes. For example, the failure of acousticians and regulatory authorities to understand and apply the mandatory assessment of “special audible characteristics” in the New Zealand standards (e.g., NZS6808:1998 and NZS6808:2010) high-lights the difficulty with measurement and assessment of anything other than very basic “noise-numbers”. In contrast, the Queensland approach applies a defined regulatory philosophy (environmental vales) and measurable acoustic quality objectives.

Shepherd *et al.* in the companion paper “Do quiet areas afford greater health-related quality of life than noisy areas?” [25] present exploratory data indicating that, relative to noisier areas, quiet areas facilitate restoration of, or impede insult to, health as reflected by HRQOL measures. Modern living is challenging, and managing stress is essential to health and wellbeing. Research is increasingly showing that disagreeable soundscapes can induce annoyance or sleep disruption, whilst positively evaluated soundscapes can aid restoration. The results reported add to the small number of studies offering quantitative evidence of the benefits afforded by quiet areas. Given the value that individuals’ place on green and quiet areas, even when located within city limits, and the possible restorative features of these areas, legislation such as that developed in Queensland can have a significant positive effect for amenity and wellbeing.

**Figure 1:**
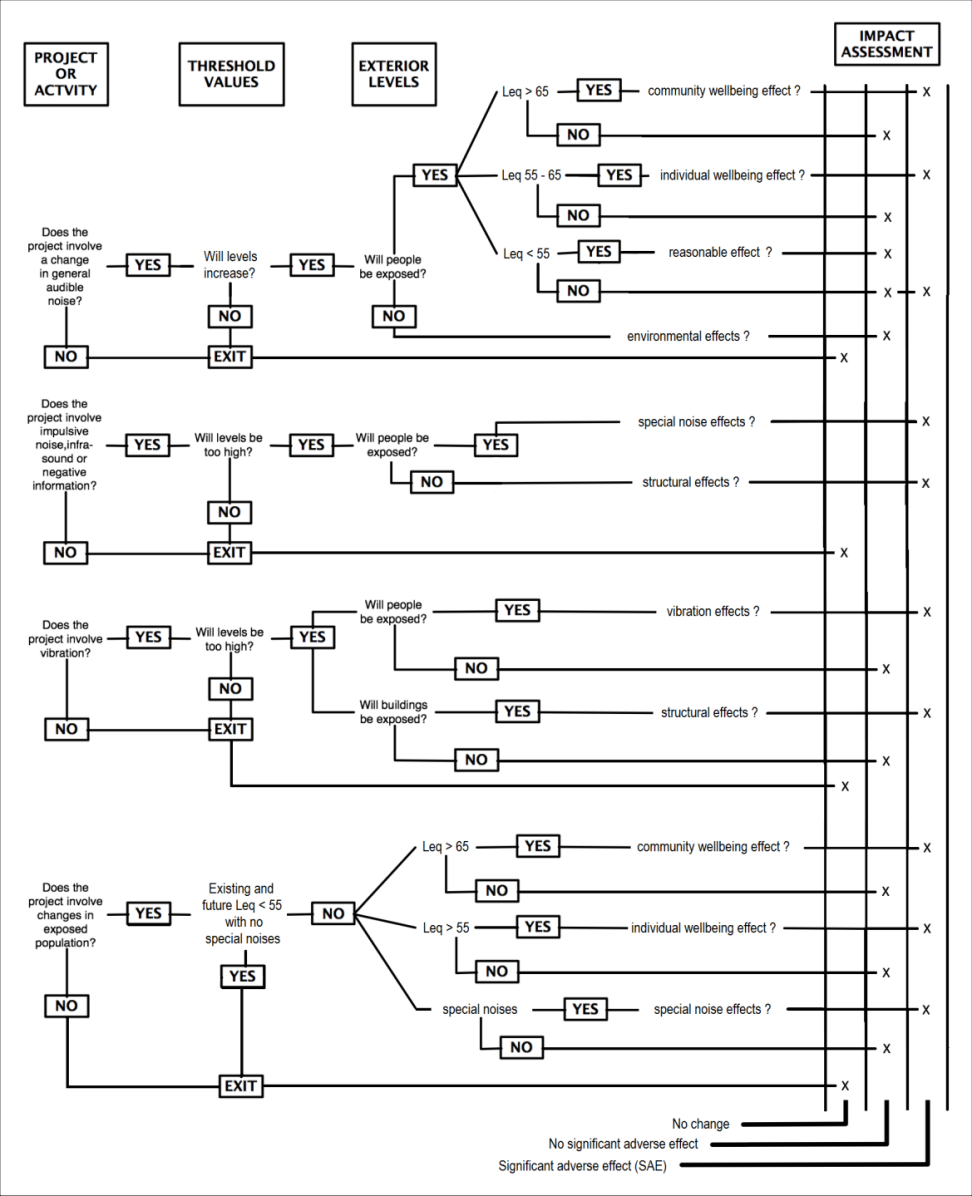
Decision processes to establish adverse effects due to noise [52,58]. This approach is adopted by the Queensland legislation.

The subjective processes a person may consciously and/or subconsciously apply when listening to a sound are described in a noise impact assessment methodology developed by the United States Environmental Protection Agency (USEPA) [58,59]. The methodology [58] is incorporated into the Queensland Environmental Protection (Noise) Policy 1997 User’s Guide [52], reproduced here in Figure 1. The “level” of noise is established in terms of fact and degree, and introduces a degree of objectivity to the process in assessing soundscapes and wellbeing. The relevance of the decision processes in Figure 1 is that they provide standard methodologies to test for noise and vibration. Consequently they provide a framework to consider “quietness” through all aspects of “noise” and the characterisation of the relevant soundscapes. The methodologies also allow the determination of baselines for the consideration of amenity and wellbeing as these apply to communities and individuals. In any situation, site-and industry-specific factors would influence the total noise reductions required to achieve compliance with sound level or potential “nuisance-abatement” criteria.

In contrast, the New Zealand approach begins with amenity but, through the use of environmental standards, ends with simply sound level criteria which have no relation to subjective factors. Unlike Queensland, the interplay between subjective and objective factors present in Figure 1 is not applied.

## 6. Conclusions

We present a description of legislative approaches taken in Queensland (Australia) and New Zealand to define wellbeing and amenity in acoustical terms. The definitions are not simple, and are not easily translated into the rigid physical structures required for acoustical measurements, nor for the black-and-white criteria that assist legal judgements. Quiet is a readily defined environmental value and determinant of good health and wellbeing under Queensland’s environmental noise legislation. In practice, wellbeing and amenity can be described in terms of sound character or quality as well as physical terms such as sound level. Queensland legislation [28] provides clear guidelines for quiet in terms of soundscape, community amenity and human wellbeing with respect to noise.

The New Zealand planning legislation [27], however, does not provide the same level of guidance with respect to soundscapes, amenity and wellbeing, as Queensland and relies heavily on local planning instruments that apply sound levels which are variable in application. New Zealand planning legislation is not structured on protecting environmental values and the Queensland approach is preferred because of its emphasis on wellbeing and amenity, and defined environmental legislative structure.
